# Exogenous Nucleotides Improve the Skin Aging of SAMP8 Mice by Modulating Autophagy through MAPKs and AMPK Pathways

**DOI:** 10.3390/nu16121907

**Published:** 2024-06-17

**Authors:** Rui Fan, Ying Zhang, Rui Liu, Chan Wei, Xiujuan Wang, Xin Wu, Xiaochen Yu, Zhen Li, Ruixue Mao, Jiani Hu, Na Zhu, Xinran Liu, Yong Li, Meihong Xu

**Affiliations:** 1 Department of Nutrition and Food Hygiene, School of Public Health, Peking University, Beijing 100191, China; fanruirf@bjmu.edu.cn (R.F.); 2011110189@bjmu.edu.cn (Y.Z.); liuruipku@163.com (R.L.); chanwei2018@126.com (C.W.); wangxiujuan076@pku.edu.cn (X.W.); wuxin12@bjmu.edu.cn (X.W.); 1410306228@pku.edu.cn (X.Y.); lizhenbjmu@163.com (Z.L.); rx334@163.com (R.M.); hujiani95@163.com (J.H.); summer920503@163.com (N.Z.); liuhappy07@163.com (X.L.); liyong@bjmu.edu.cn (Y.L.); 2 Beijing Key Laboratory of Toxicological Research and Risk Assessment for Food Safety, Peking University, Beijing 100191, China

**Keywords:** exogenous nucleotides, skin aging, SAMP8 mice, autophagy, AMPK pathway, MAPK pathway

## Abstract

The skin, serving as the body’s primary defense against external elements, plays a crucial role in protecting the body from infections and injuries, as well as maintaining overall homeostasis. Skin aging, a common manifestation of the aging process, involves the gradual deterioration of its normal structure and repair mechanisms. Addressing the issue of skin aging is increasingly imperative. Multiple pieces of evidence indicate the potential anti-aging effects of exogenous nucleotides (NTs) through their ability to inhibit oxidative stress and inflammation. This study aims to investigate whether exogenous NTs can slow down skin aging and elucidate the underlying mechanisms. To achieve this objective, senescence-accelerated mouse prone-8 (SAMP8) mice were utilized and randomly allocated into Aging, NTs-low, NTs-middle, and NTs-high groups, while senescence-accelerated mouse resistant 1 (SAMR1) mice were employed as the control group. After 9 months of NT intervention, dorsal skin samples were collected to analyze the pathology and assess the presence and expression of substances related to the aging process. The findings indicated that a high-dose NT treatment led to a significant increase in the thickness of the epithelium and dermal layers, as well as Hyp content (*p* < 0.05). Additionally, it was observed that low-dose NT intervention resulted in improved aging, as evidenced by a significant decrease in p16 expression (*p* < 0.05). Importantly, the administration of high doses of NTs could improve, in some ways, mitochondrial function, which is known to reduce oxidative stress and promote ATP and NAD^+^ production significantly. These observed effects may be linked to NT-induced autophagy, as evidenced by the decreased expression of p62 and increased expression of LC3BI/II in the intervention groups. Furthermore, NTs were found to upregulate pAMPK and PGC-1α expression while inhibiting the phosphorylation of p38MAPK, JNK, and ERK, suggesting that autophagy may be regulated through the AMPK and MAPK pathways. Therefore, the potential induction of autophagy by NTs may offer benefits in addressing skin aging through the activation of the AMPK pathway and the inhibition of the MAPK pathway.

## 1. Introduction

Skin, the largest organ of the human body, has essential functions in protection, temperature regulation, sensation, secretion, excretion, immunity, and homeostasis maintenance [1]. As the primary external barrier of the body, the skin bears the traces of time and life. The skin undergoes visible signs of aging due to both intrinsic and extrinsic factors, manifesting as wrinkles, age spots, dryness, thinning, and reduced elasticity [2]. Skin aging is not solely a matter of aesthetics, but also poses a significant health challenge. It can manifest as the aging phenotype in other tissues and organs, contributing to overall bodily aging [3]. Additionally, skin aging serves as a reflection of internal health. With a decrease in the rate of epidermal renewal, compromised barriers, and diminished wound healing quality, older adults are at an elevated risk for infections and chronic diseases, including cognitive impairments and cardiovascular diseases [4,5]. Consequently, a thorough comprehension of skin aging is essential for a comprehensive understanding of overall health, mortality risk, and longevity [6,7].

Skin aging is influenced by a combination of intrinsic and extrinsic factors. Intrinsic aging is a natural physiological progression primarily driven by genetic or metabolic factors, whereas extrinsic aging is caused by external environmental factors such as air pollution, smoking, inadequate nutrition, and sun exposure [8]. Various models have been put forth to elucidate the molecular mechanisms underlying skin aging, encompassing theories such as cellular senescence, diminished cellular DNA repair capabilities, telomere attrition, extranuclear mitochondrial DNA point mutations, oxidative stress, heightened incidence of chromosomal abnormalities, single-gene mutations, reduced glycation, chronic inflammation, among others [9].

Skin aging is characterized by a reduction in the efficacy of the endogenous antioxidant system, leading to the increased formation of reactive oxygen species (ROS) that primarily cause DNA damage [10]. ROS have the potential to trigger premature cellular senescence, which is believed to contribute to skin aging and age-related diseases. Mitochondria, as both generators and recipients of oxidative stress, are particularly vulnerable to the accumulation of somatic mitochondrial DNA mutations resulting from ROS exposure [11]. Mitochondria serve as the primary source of cellular energy, playing a crucial role in sustaining cell viability and tissue metabolism. The process of mitochondrial biogenesis, which involves the generation of new and functional mitochondria, is regulated by various signaling cascades. PGC-1α is recognized as the key regulator of mitochondrial biogenesis, with elevated levels of PGC-1α promoting the generation of new mitochondria, enhancing mitochondrial function, and delaying the aging process induced by intrinsic and extrinsic factors on the skin [12,13]. Consequently, the process of mitochondrial biogenesis plays a crucial role in skin aging [14]. Moreover, autophagy is regarded as a pivotal process in the regulation of skin aging. It has been demonstrated to eliminate senescent subcellular organelles and proteins, as well as to govern the pigmentation, equilibrium, and activities of keratinocytes, skin dermal fibroblasts, and melanocytes in response to both external and internal factors, thereby facilitating the aging of the skin [15,16]. The MAPK signaling pathway has been identified as a potential intersection point between oxidative stress-induced skin aging and autophagy [17]. Consequently, a comprehensive understanding of the mechanisms underlying skin aging may facilitate the identification of preventative and therapeutic interventions aimed at decelerating the aging process of the skin [18].

Emerging strategies for addressing aging involve the elimination of senescent cells through the use of senolytic drugs such as dasatinib, metformin, rapamycin, and fisetin. However, dasatinib is not specific to skin-related anti-aging methods, and metformin, rapamycin, and fisetin have not been tested in population trials) [19,20,21,22]. In addition, the reasonable local application of antioxidants is an effective protection strategy against skin aging including some pharmacological drugs, antioxidants and active chemical compounds such as CoQ10 [23], ginsenoside RG3 [24], nicotinamide [25], HCP1 [26], h hydroxyproline [27], mitochondria-targeted H_2_S [28]. Nucleotides (NTs) are fundamental components of nucleic acids and play crucial roles in various intricate physiological processes such as energy metabolism, intercellular signal transduction, enzymatic reactions, and cell cycle regulation as a synthetic nitrogen source. NTs are considered essential nutrients in specific circumstances, including disease, intestinal injury, immune challenge, starvation, aging, rapid growth and development, limited nutrient intake, and endogenous synthesis and expression, despite the body’s ability to absorb and utilize them from the diet [29,30]. Extensive research has confirmed the health benefits of exogenous NTs, including antioxidant activity, immunomodulatory activity, DNA protective activity, the promotion of cell proliferation, the maintenance of liver and intestinal function [31]. Recent work performed in our laboratory revealed that exogenous NTs are responsible for the restoration of mitochondrial function and the augmentation of mitochondrial ATPase [32], are involved in the synthesis of NAD^+^, ATPapae, consequently leading to the promotion of mitochondrial biogenesis via the NAD^+^/SIRT1/PGC-1α pathway in the H_2_O_2_-induced senescent NIH/3T3 cell model [33]. Furthermore, our team has determined that NTs exhibit a higher level of antioxidant activity compared to the widely recognized anti-aging drug nicotinamide mononucleotide (NMN) [34]. Taken together, these findings indicate that exogenous nucleotides (NTs) may hold promise for exerting anti-aging effects on the skin. Nevertheless, there is a notable gap in the existing literature regarding the potential senescence-ameliorating properties of NTs. Furthermore, limited research has been conducted on the anti-aging effects of NTs, despite their relatively safe profile as natural components of the body in comparison to pharmaceutical drugs and certain chemicals.

This study examined the impact of NTs on skin aging in senescence-accelerated mouse prone-8 (SAMP8) mice to investigate the potential benefits of NTs in mitigating senescence and elucidate their anti-aging mechanisms. The findings offer insights into the development of a safer nutritional intervention strategy for preventing and treating skin aging.

## 2. Materials and Methods

### 2.1. Chemicals

The NTs, 5′-guanosine monophosphate disodium salt(GMP), 5′-disodium uridine-monophosphate (UMP), 5′-cytimidine monophosphate(CMP), and 5′-adenosine monophosphate (AMP), are produced from sucrose molasses by enzymatic degradation with a purity of >99%. The exogenous NT mixture used in this current study was prepared with the ratio as follows, 5′AMP:5′CMP:5′GMPNa2:5′UMPNa2 = 16:41:19:24. All of them were supplied by Hainan Shuangdi Zhen-Ao Life Science Research Center Co. Ltd. (Dalian, China).

### 2.2. Animals and Treatment

**Figure 1:**
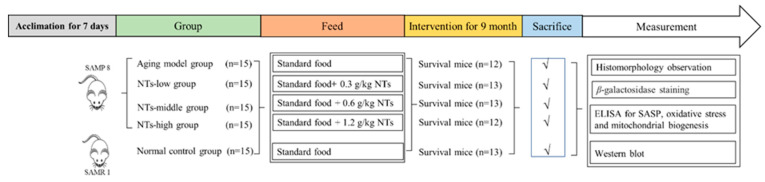
The flow of mouse treatments.

The SPF SAMP8 and SAMR1 mice (3-month-old males) were procured from the Department of Experimental Animal Science at Peking University and were subsequently housed individually in barrier-grade animal rooms with a 12 h alternating light cycle. The temperature was 24 ± 2 °C, and relative humidity was 50–60%. The mice were allowed to feed and drink freely. After 1 week of feeding for adaptation, all SAMP8 mice were grouped as follows: aging model (Aging), NTs-low (NTs-L), NTs-middle (NTs-M), and NTs-high (NTs-H) groups of 15 mice. Meanwhile, SAMR1 mice were set as the normal control group (Normal, n = 15). The Aging and Normal groups were provided with standard food (American Institute of Nutrition Rodent Diets-93M), while varying doses of exogenous NTs were incorporated into the standard food for the three NT-intervention groups. Detailed information regarding the groups, diet, and number of animals can be found in Figure 1. After a 9-month intervention, some of the dorsal skin tissue underwent partial pathological testing and others was subsequently collected and stored at −80 °C until Western blot analysis was conducted.

### 2.3. Histomorphology Observation

#### 2.3.1. Hematoxylin-Eosin Staining

The dorsal skin tissues of mice were fixed in 4% paraformaldehyde for 48 h, decalcified in 15% EDTA for 2 weeks at room temperature, and subsequently embedded in paraffin. Sections were then prepared and stained with hematoxylin-eosin (HE). Observations were made using a light microscope and analysis was conducted using ImageJ software (Image pro plus 6.0) (BX43F, Olympus, Tokyo, Japan).

#### 2.3.2. Two-Photon Excitation Fluorescence Imaging

Based on previous reports [35,36], the epidermal and dermal layers were visualized using the LSM510 confocal laser microscope (Zeiss Corporation, Oberkochen, Germany) equipped with the femtosecond lasers Verdi-10 and Mira-900 (Coherent Corporation, Palo Alto, CA, USA). The skin samples were freshly excised and sectioned perpendicular to the epidermal layer to ensure that each slice encompassed a comprehensive transverse representation of both the epidermal and dermal layers. The tissue sections were left unstained and affixed to glass slides for microscopic TPEF and SHG imaging. The SHG detection channel was set to 1 at 425 ± 20 nm, while the TPEF detection channel was set to 2 at 450–714 nm. An excitation light source of 850 nm was utilized, with an output power of less than 10 mW. Image capture was set at 512 pixels × 512 pixels.

### 2.4. Senescence-Associated-β-Galactosidase Staining

Pre-embedding senescence-associated-β-galactosidase (SA-β-gal) staining was performed as previously described. Briefly, fresh skin from the dorsum were incubated in the β-galactosidase staining solution at 37 °C overnight, and then fixed with PLP (2% paraformaldehyde containing 0.075 M lysine and 0.01 M sodium periodate) for 24 h. Following the dehydration and embedding in paraffin, samples were cut into 5 μm sections. The dewaxed sections were counterstained with nuclear fast red and mounted.

### 2.5. ELISA Analysis 

#### 2.5.1. Senescence-Associated Secretory Phenotype (SASP) Analysis

Senescence-associated secretory phenotypes (SASP) including interleukin-1β (IL-1β), interleukin-6 (IL-6), matrix metalloproteinase-3 (MMP-3), and vascular cell adhesion molecule 1 (VCAM-1) were measured with ELISA analysis. A 10% tissue homogenate was prepared for test kit detection. The IL-1β, IL-6 assay kit were obtained from Invitrogen Co., Ltd. (Waltham, MA, USA), the MMP-3 assay kit was obtained from R&D Biotechnology Co., Ltd. (Minneapolis, MN, USA), VCAM-1 assay kit was obtained from Miltiscience Co., Ltd. (Hangzhou, China). All of these indicators were measured according to the protocols provided with the assay kits.

#### 2.5.2. The Oxidative Stress-Associated Biological Indicators Analysis

The oxidative stress-associated biological indicators including malondialdehyde (MDA) and total superoxide dismutase (SOD) were measured with ELISA analysis. A 10% tissue homogenate was prepared for test kit detection. MDA and SOD assay kits were purchased from the Nanjing Jiancheng Bioengineering Institute. The biological indicators were measured according to the protocols provided with the assay kits.

#### 2.5.3. The Mitochondrial Function-Associated Biological Indicators Analysis

The mitochondrial function, including ATP and NAD^+^/NADH. NAD^+^/NADH, was analyzed using the assay kit with WST-8, obtained from Beyotime Co., Ltd. (Shanghai, China), and ATP assay kit from the Nanjing Jiancheng Bioengineering Institute (Nanjing, China). 

#### 2.5.4. Hydroxyproline (Hyp) Content Analysis

The Hyp level of skin was detected and performed using hydroxyproline assay kit (Nanjing JianCheng, Nanjing, China) according to the kit instructions. Briefly, the skin was weighed and subsequently boiled in a water bath at 95 °C for 20 min along with the hydrolysate solution. The pH of the hydrolysate solution was adjusted to a range of 6.0 to 6.8. Following dilution of the hydrolysate, the supernatant was obtained through centrifugation. The supernatant samples were utilized for detection, with double-distilled water serving as the blank control. Samples and blank, standard solution were introduced into the test tube, followed by the addition of Reagent I at room temperature for a duration of 10 min. Subsequently, Reagent II was added at room temperature for 5 min. Next, Reagent III was added for a period of 15 min at 60 °C, followed by centrifugation after cooling. Finally, the supernatants were analyzed using a Multiskan Microplate Reader (Thermo Fisher, Waltham, MA, USA) at a wavelength of 550 nm.

### 2.6. Western Blot Analysis

Three mice from each group were randomly chosen for Western blot analysis. Total proteins were extracted and quantified using a BCA Protein Assay Kit. The lysates were separated by gel electrophoresis and transferred to PVDF membranes. A solution of 5 g of skimmed milk powder in 100 mL TBST was used as a blocking agent for 4 h at room temperature. Following blocking, the membranes were exposed to primary antibodies overnight at 4 °C.

Protein expression was detected using a primary antibody, p16INK4A Rabbit pAb (1:500, Abclonal, Wuhan, China), phospho-p38 MAPK (Thr180/Tyr182) (D3F9) Rabbit mAb(1:1000, CST, Danvers, MA, USA), Phospho-SAPK/JNK (Thr183/Tyr185) (81E11) Rabbit mAb (1:1000, CST, Danvers, MA, USA), Phospho-p44/42 MAPK (Erk1/2) (Thr202/Tyr204) XP Rabbit mAb (1:1000, CST, Danvers, MA, USA), Phospho-AMPKa (Thr172) (40H9) Rabbit mAb (1:1000, CST, Danvers, MA, USA), ULK2 Rabbit pAb (1:500 Abclonal, Wuhan, China), SQSTM1/p62 Antibody (1:1000, CST, Danvers, MA, USA), LC3B (E7X4S) XP Rabbit mAb (1:1000, CST, Danvers, MA, USA), Anti-PGC1 alpha Antibody (1:1000, Abcam, Cambridge, UK). Goat Anti-Rabbit IgG H&L secondary antibodies (1:10,000, Abcam, Cambridge, UK). Anti-beta actin antibody was obtained from Abcam(Cambridge, UK), Quantitative analysis of Western blot was performed using Image-Pro Plus (Media Cybernetics, Rockville, MD, USA).

### 2.7. Statistical Analysis

Statistical analyses were conducted utilizing SPSS software version 24 (SPSS Inc., Chicago, IL, USA). The data were presented as mean ± standard deviation (SD) and subjected to one-way analysis of variance (ANOVA) testing; the distinction of parametric samples among groups was assessed through multiple comparisons utilizing the least significant difference (assuming equal variances) or Dunnett’s T3 test (not assuming equal variances). A *p*-value of less than 0.05 denoted a statistically significant difference.

## 3. Results

### 3.1. The Effect of NTs on the Morphology

**Figure 2:**
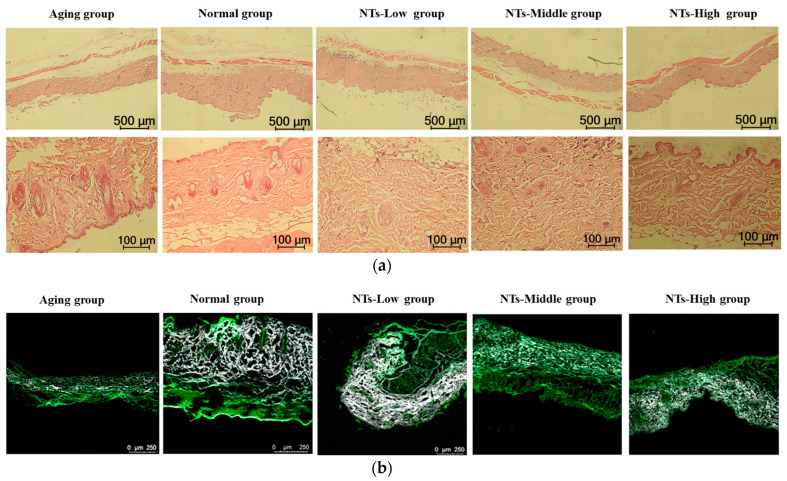

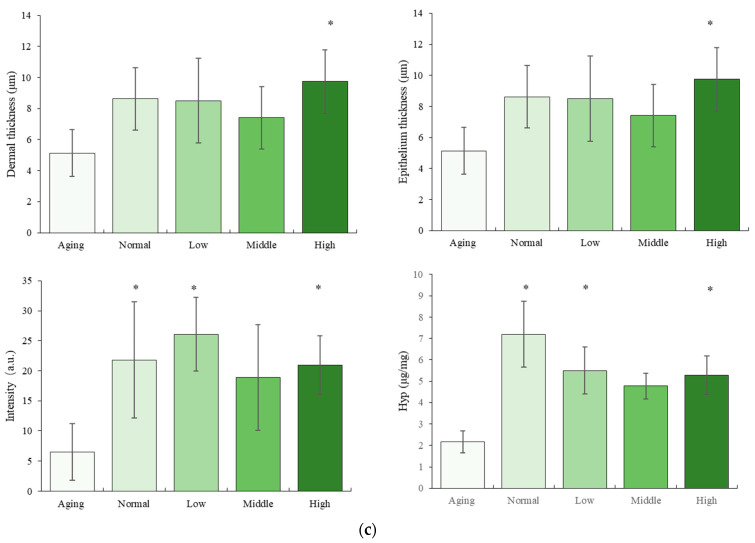
The morphology and structure of mice skin in different groups. (**a**) H&E staining; (**b**) two-photon excitation fluorescence imaging (white, collagen fiber; green, NADH); (**c**) the thickness, fluorescence intensity and hydroxyproline content; all experimental data are displayed as mean ± SD. * *p* < 0.05 versus Aging group.

The skin was stained with hematoxylin and eosin (HE) and its morphology was examined using an optical microscope (Figure 2a). In the control group, distinct boundaries were observed between the basal layer, the acanthocyte layer, the granular layer, and the cuticle. The dermis, situated beneath the epidermis and housing blood vessels, sweat glands, hair follicles, and sebaceous glands, exhibited impaired skin tissue growth in the aging model group. As time progressed, the dermis experienced significant thinning and loosening, the epidermal–dermal junction became flattened, and the adhesion between the epidermis and dermis noticeably decreased. In contrast to the Aging group, the dermis in the intervention groups exhibited a higher degree of compactness. Specifically, the high-dose-NT group demonstrated normal skin tissue morphology characterized by a regular arrangement of epidermal cells and intact dermal structures. The findings presented in Figure 2c demonstrate the dermal and epidermal thickness of mice in each experimental group. In comparison to the Aging group, the Normal and the intervention groups, particularly those receiving a high dose of NTs, exhibited a significant increase in dermal and epidermal thickness (*p* < 0.05). These results suggest that NTs have the potential to delay the thinning of the dermal and epidermal layers, with a more pronounced effect observed at higher doses of NTs.

The fluorescence signal intensity of collagen fibers was detected by TPEF imaging technology in the current study, and the results are shown in Figure 2b,c. In comparison to the control group, the Aging group exhibited a reduction in skin collagen fiber density and fluorescence signal intensity, with statistical significance (*p* < 0.05). Conversely, the Normal group and NT-intervention groups displayed dense, robust, and plentiful skin collagen fibers, with a significant increase in fluorescence signal intensity in both low and high intervention groups (*p* < 0.05). These findings suggest that NT intervention led to the repair of skin collagen fibers in mice. Furthermore, the Hyp content in the Aging group was found to be significantly lower compared to the Normal group. Following the administration of NTs, there was a significant increase in Hyp content (*p* < 0.05), suggesting that NTs may have the ability to enhance collagen fiber production.

### 3.2. The Anti-Aging Effects of NTs

**Figure 3:**
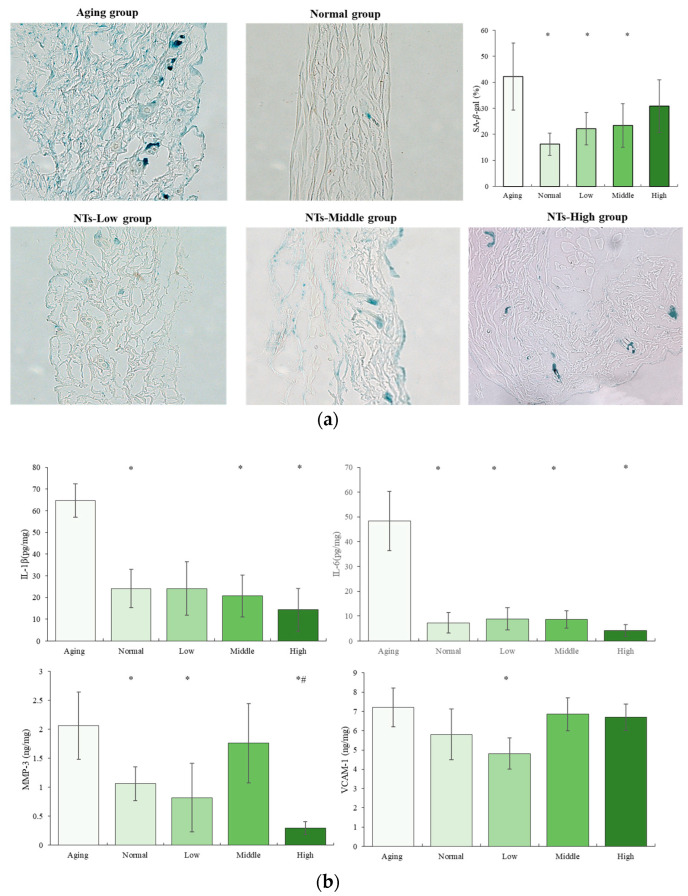
The anti-aging effects of NTs. (**a**) The SA-β-gal activity; (**b**) the lever of SASP. All experimental data are displayed as mean ± SD. # *p* < 0.05 versus Normal group, * *p* < 0.05 versus Aging group.

SA-β-gal is a frequently utilized marker of cellular senescence. A notable elevation in SA-β-gal activity was noted in the Aging group in comparison to the control group. Conversely, a significant reduction in the number of SA-β-gal-positive cells was observed following the administration of low and middle NTs when compared to the Aging group (*p* < 0.05) (Figure 3a). The secretion of senescence-associated secretory phenotype (SASP) was another common senescence marker. As demonstrated in Figure 3b, the concentrations of IL-1β, IL-6, and MMP-3 in the Aging group exhibited a significant increase compared to the Normal group (*p* < 0.05), suggesting that the aging SAMP8 mice were experiencing an inflammatory state. Following intervention with middle and high doses of NTs, the secretion of IL-1β significantly decreased (*p* < 0.05), while the secretion of IL-6 significantly decreased in all NT-intervention groups (*p* < 0.05). A clear dose–response relationship was observed in the inhibitory effect on IL-1β and IL-6 secretion in aging SAMP8 mice, with a greater effect seen with higher intervention doses. In contrast, MMP-3 levels exhibited a different response, with significant decreases observed after intervention with both low and high doses of NTs compared to the Aging group (*p* < 0.05). Furthermore, the high dose intervention group showed significantly lower MMP-3 levels compared to the Normal group (*p* < 0.05). In comparison to the Aging group, the Normal group and all intervention groups exhibited relatively lower levels of VCAM-1. Specifically, the low-dose NT group demonstrated significantly lower levels of VCAM-1 compared to the other groups (*p* < 0.05).

### 3.3. The Effects of NTs on Antioxidant Activity and Mitochondrial Biogenesis

**Figure 4:**
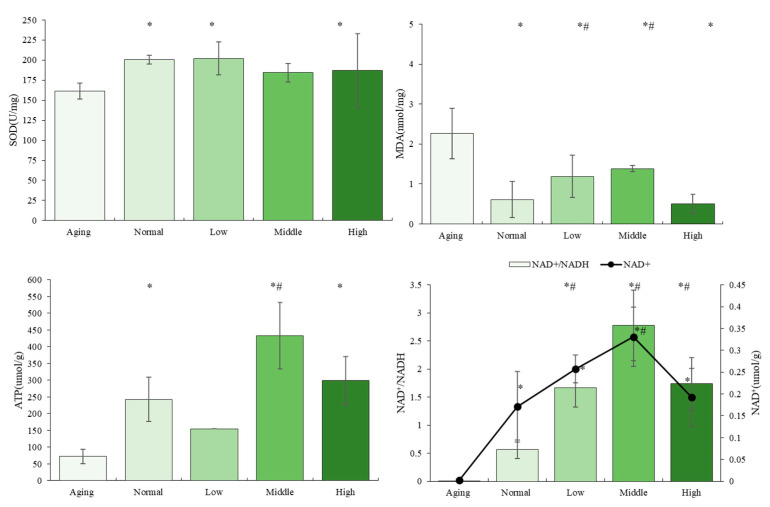
The effects of NTs on the antioxidant activity and mitochondrial biogenesis. All experimental data are displayed as mean ± SD. # *p* < 0.05 versus Normal group, * *p* < 0.05 versus Aging group.

As depicted in Figure 4, the aging SAMP8 mice exhibited a marked decrease in the activities of superoxide dismutase (SOD) and a significant increase in malondialdehyde (MDA) levels (*p* < 0.05). In comparison to the Aging group, SOD activities significantly increased in both the low- and high-dose-NT groups (*p* < 0.05), whereas MDA levels were significantly lower in all dose-intervention groups. A reduction in ATP production was observed in the Aging group compared to the control group. Significantly higher levels of ATP production were noted in the middle- and high-dose groups of NT intervention (*p* < 0.05), with the middle-dose intervention group exhibiting notably higher ATP production levels than the control group (*p* < 0.05).

Figure 4 illustrates that there were no significant differences in NAD^+^/NADH levels between the Normal and Aging groups (*p* > 0.05). However, NAD^+^ levels in the Aging group were notably lower compared to the other groups (*p* < 0.05). Following intervention with NTs, there was a significant increase in NAD^+^ levels and NAD^+^/NADH levels (*p* < 0.05). Even low and moderate doses of NTs were able to significantly elevate NAD^+^ levels and NAD^+^/NADH levels when compared to the Normal group (*p* < 0.05).

### 3.4. The NTs Improve Aging and May Activate Autophagy

The Aging group exhibited a significantly higher expression of p16 compared to the non-treated (NT) intervention group with a low dose. However, there was no significant alteration in the expression of p16 in the low-dose NT groups. Conversely, the high-dose NT groups demonstrated a significantly higher expression of p16 (*p* < 0.05). There was no significant difference in the expression of LC3BI/II between the Aging and Normal groups (*p* > 0.05) prior to intervention. However, following intervention, there was a significant increase in the expression of LC3BI/II (*p* < 0.05), with p62 expression being significantly higher in all four groups (*p* < 0.05). Additionally, the administration of NTs was found to decrease the expression of p62, suggesting that NT intervention may activate autophagy.

### 3.5. NTs Activate the AMPK Pathway and Inhibit the MAPK Pathway

The Aging group exhibited a higher expression of pAMPK, which was subsequently upregulated following intervention with NTs (*p* < 0.05). While there was an increasing trend in ULK2 expression among the intervention groups, no significant differences in ULK2 expression were observed between the Aging group and the Normal group (*p* > 0.05). However, following intervention with a high dose, ULK2 expression significantly increased (*p* < 0.05).

Following intervention with NTs, a notable increase in PCG-1α expression was observed (*p* < 0.05), consistent with the observed trend. Furthermore, a significant elevation in the expression of *p*-p38, *p*-ERK, and *p*-JNK was noted in the Aging group compared to the Normal group (*p* < 0.05). Following the intervention, a statistically significant reduction was observed in the levels of *p*-p38, *p*-ERK, and *p*-JNK (*p* < 0.05).

## 4. Discussion

This study examined the impact of NTs on skin aging in SAMP8 mice in order to investigate their potential efficacy in ameliorating age-related changes. Traditional rodent models typically require at least 18 months to exhibit aging phenotypes, with a 24-month-old normal mouse considered to be roughly equivalent to an 80-year-old human based on average life spans [37]. However, the SAMP8 mouse model accelerates the aging process [38]. The average life span of SAMP mice is approximately 40% shorter than that of normal mice [39]. The senescence accelerated mouse (SAM) is a group of inbred mouse strains that have been developed through the backcrossing of AKR/J mice and subsequent phenotyping. This model is utilized for the study of human aging and age-related diseases, such as cognitive disorders and osteoporosis [40,41]. It was determined that the expression levels of several genes involved in regulating skin function, such as Ela, Flg, Lor, Col1a1, Col1a2, and aquaporin-3, were reduced in 8-month-old SAMP1 mice compared to SAMR1 mice, which indicated SAMP mice could be a tool for analyzing skin aging [42]. In this research, 12-month-old SAMP mice were utilized as a model organism, with an approximate age equivalence to 70 years in humans [38], to investigate the potential anti-aging properties of NTs and elucidate their underlying mechanisms. The application of senolytics topically to target skin senescence may offer promising avenues for the advancement of innovative anti-aging interventions, potentially delaying systemic aging processes and the manifestation of age-related illnesses.

In this current study, we first found that NTs improved skin morphology and structure in aging mice. From Figure 2, a significantly thinner and looser dermis was observed in the Aging group, while, the dermises in the intervention groups were more tightly packed. The high-dose-NT group showed normal skin tissue morphology with a regular epidermal cell layer arrangement and normal dermal integral structures. Compared with the Aging group, the dermal and epidermal thickness in the Normal and intervention groups were increased, of which those from the high-dose NT-intervention group were significantly increased (*p* < 0.05); meanwhile, the skin collagen fibers of the Normal group and NT-intervention groups were dense, strong and abundant; the fluorescence signal intensity of the collagen fibers in the low- and high-intervention groups was significantly increased (*p* < 0.05). Research finds that as a person ages, the proliferation of cells in the basal layer reduces. The epidermis then becomes thinner, and the contact surface area between dermis and epidermis decreases, resulting in a smaller exchange surface for nutrition supply to the epidermis and further weakened ability of basal cell proliferation [43]. Therefore, intervention with NTs could improve skin aging.

Aging is characterized as a gradual physiological deterioration that results in frailty, age-related ailments, and eventual mortality. Cellular senescence, an adaptive response to harmful stimuli, has been identified as a key feature of the aging process, leading to diminished tissue functionality in advanced age. Senescent cells in the skin display characteristic markers of cellular senescence. The biomarkers of skin aging are becoming more commonly utilized in clinical settings to assess the physiological alterations in aging skin and aging-associated conditions, as well as to accurately discern the impacts of interventions. One of the earliest and most commonly utilized markers for cellular senescence is SA-β-Gal, which continues to be the benchmark for detecting senescent cells both in vitro and in tissues. The analysis of Figure 3 reveals a notable decrease in SA-β-gal-positive cells following treatment with low and moderate levels of NTs, as compared to the Aging group (*p* < 0.05).

**Figure 5:**
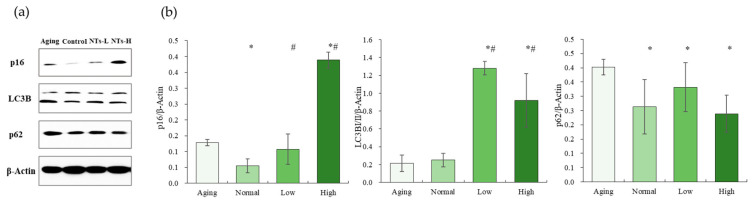
The effects of NTs on the protein expressions of autophagy. (**a**) Protein expressions of autophagy; (**b**) the Western blot strip (n = 3 per group). All experimental data are displayed as mean ± SD. # *p* < 0.05 versus Normal group, * *p* < 0.05 versus Aging group.

Cellular senescence is a state of stable cell-cycle arrest marked by the presence of an inflammatory phenotype referred to as the senescence-associated secretory phenotype (SASP) [44], along with the accumulation of oxidative stress-induced damage [45], telomere shortening, and mitochondrial dysfunction [46]. The senescence-associated secretory phenotype (SASP) is characterized by the secretion of pro-inflammatory cytokines (such as IL-1, IL-6, and IL-8), growth factors (including HGF, GRO, and TGF-β), insulin-like growth factor-binding protein 7 (IGFBP-7), chemokines (such as CXCL-1/3, CXCR2), and MMPs [47] Our study revealed a significant increase in the concentrations of IL-1β, IL-6, and MMP-3 in the Aging group compared to the Normal group (*p* < 0.05), consistent with previous findings. Following NT intervention, there was a significant decrease in IL-1β and IL-6 levels, displaying a regular dose–response relationship, as illustrated in Figure 3b. MMPs play a crucial role in cell communication and are responsible for cell–matrix signaling events. As a class of proteolytic enzymes, their primary physiological function is to degrade various protein components in the extracellular matrix (ECM). Previous research has shown that high levels of MMPs were detected in both SAMP8 and SAMR1 mice at 12 months of age [48]. This similar phenomenon was observed in the Aging group, with a corresponding significant decrease in Hyp content. However, NT intervention was found to ameliorate these effects. The decrease in collagen synthesis by fibroblasts in aged skin can lead to the deterioration of the structural integrity of the skin’s extracellular matrix, resulting in diminished intercellular adhesion and destruction of the cytoskeletal structure [49]. The upregulation of intercellular adhesion molecule ICAM-1 and vascular cell adhesion molecule VCAM-1 has been observed in patients with atopic dermatitis, even in seemingly healthy skin [50]. Similarly, our study revealed a significant increase in VCAM-1 levels in aging SAMP8 mice and SAMR1 mice, with a notable decrease following low-dose NT treatment. These findings suggest that NTs may have a beneficial effect on inflammatory skin disorders in aging skin, consistent with previous research [51]. So far, our research has demonstrated that NT intervention has the potential to ameliorate the skin-aging phenotype in aging mice, as well as diminish the secretion of senescence-associated secretory phenotype (SASP) in the skin of aging mice. Furthermore, we hypothesize that the improvement in skin aging observed with NTs may be attributed to its ability to exhibit cell senescence effects, as evidenced by the reduced protein expression of p16 (see Figure 5), a finding consistent with prior literature [32]. p16 blocks the phosphorylation of retinoblastoma (Rb), forming the E2F complex that blocks cells in the G1 phase, and is considered to be one of the universal biomarkers of cellular senescence due to its obvious changes, convenient detection, and its ability to be found in various senescent cells [52]. As a stress response, cellular senescence can force cells out of the cell cycle and loss the ability to cope with growth factors or mitogens [53]. The aging markers p16 and p21 are significantly higher in SHJHhr mouse skin than ICR mouse skin at the same age [54]. 

The researchers have demonstrated a correlation between oxidative stress, mitochondrial function, and autophagy in the process of skin aging [55,56]. Based on these evidences, it was hypothesized that the anti-aging effects of NTs may be attributed to the activation of autophagy, enhancement of mitochondrial function, and reduction of oxidative stress. This hypothesis was supported by subsequent measurements of ATP and NAD^+^ production, SOD levels, MDA content (Figure 4), as well as the expression levels of autophagy-related proteins LC2BI/II and p62 (Figure 5). At the molecular level, aged skin exhibits damaged mitochondria, mtDNA deletions, elevated levels of reactive oxygen species (ROS), and oxidative stress in both the dermal and epidermal layers. It has been hypothesized that NAD^+^ may influence the aging process by regulating DNA repair mechanisms and preventing stem cell senescence. Additionally, it is suggested that NAD^+^ may positively impact mitochondrial oxidative phosphorylation [57].

The diminishment of mitochondrial sirtuins results in the manifestation of the mitochondrial-dysfunction-associated senescence (miDAS) phenotype, which is distinguished by decreased NAD^+^/NADH ratios [58]. This decline in NAD^+^ levels leads to the suppression of mitochondrial energy production, ultimately contributing to the aging process and the development of age-related ailments [59]. The skin is characterized by a rapid turnover rate, as the epidermis undergoes continuous regeneration. Epidermal progenitor cells exhibit high levels of proliferation and metabolic activity, relying on adenosine triphosphate (ATP) for energy production. ATP is primarily generated through oxidative phosphorylation (OXPHOS) within the mitochondria, the cellular bioenergetics hub in eukaryotic cells [14]. Furthermore, autophagy has been demonstrated to regulate cellular stress and maintain NAD levels [60]. Nicotinamide mononucleotide (NMN)is a key NAD^+^ intermediate; extensive studies have shown that supplementing NMN ameliorates age-associated pathophysiologies and disease conditions [61]. While NMN is also a derivative of NTs and has a similar chemical structure, it was therefore inferred that NTs help to promote the biosynthesis pathways to produce NAD^+^ leading to autophagy to play an anti-aging role, which was accordant with the previous report [62].

**Figure 6:**
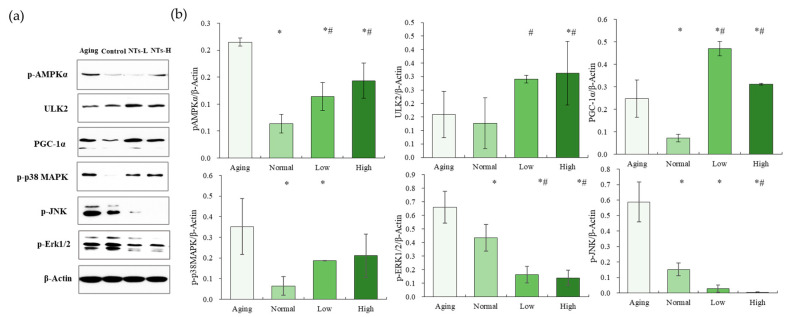
The effects of NTs on the protein expressions of MAPK and AMPK signal pathways. (**a**) Protein expressions; (**b**) the Western blot strip (n = 3 per group). All experimental data are displayed as mean ±SD. # *p* < 0.05 versus Normal group, * *p* < 0.05 versus Aging group.

How do NTs exert their anti-aging effects through autophagy and the biosynthesis of NAD^+^? We focused on p38 and the AMPK pathways in this current study. A key aspect of skin aging is the degradation of collagen, which can be induced by reactive oxygen species (ROS) through the stimulation of mitogen-activated protein kinase (MAPK) and c-Jun N-terminal kinase (JNK) signaling pathways [63,64]. The MAPK family of proteins, namely JNK, p38, and extracellular signal-regulated kinase (ERK) [65,66], was found to be expressed obviously in the Aging group, as evidenced by higher levels of MMP-3 and lower Hyp content. However, following intervention with NTs, a notable reduction in *p*-JNK and *p*-ERK expression was observed, potentially attributable to the inhibition of the p38 pathway leading to decreased MMP-3 production and subsequent collagen degradation (see Figure 6). The MAPK signaling pathway has been identified as being intricately linked to autophagy through its interaction with mTOR. Our research revealed that NT intervention can enhance autophagy, potentially through the inhibition of the p38 pathway, a phenomenon also observed with astragaloside [67].

PGC-1α serves as a crucial signaling molecule in the regulation of mitochondrial biogenesis, mitochondrial DNA replication, transcription, and translation into proteins by translation factors, as well as in response to wound injury, inflammatory agents, and UVB exposure [68]. PGC-1α has the potential to influence epidermal stem cell fate and skin repair through the maintenance of the nicotinamide adenine dinucleotide (NAD) metabolism [69]. The expression and activation of PGC-1α are governed by the AMPK/PGC-1α pathway [34]. Numerous natural extracts have been shown to enhance the expression of PGC-1α in the treatment of intrinsic skin aging, thereby facilitating mitochondrial biogenesis and retarding the aging process [13,70]. The intervention of NTs in this study resulted in a significant increase in the expression of PGC-1α, leading to enhanced NAD^+^ production, potentially through the activation of the AMPK pathway, despite the high expression of *p*-AMPK observed in the Aging group. Our research team also observed a significant increase in brown adipose tissue expression in aging SAMP8 mice, and noted that low-dose NT intervention led to an increase in *p*-AMPK expression [34]. It is hypothesized that the abnormal phenomenon of high expression in the Aging group may be attributed to reduced ATP production [71].

**Figure 7:**
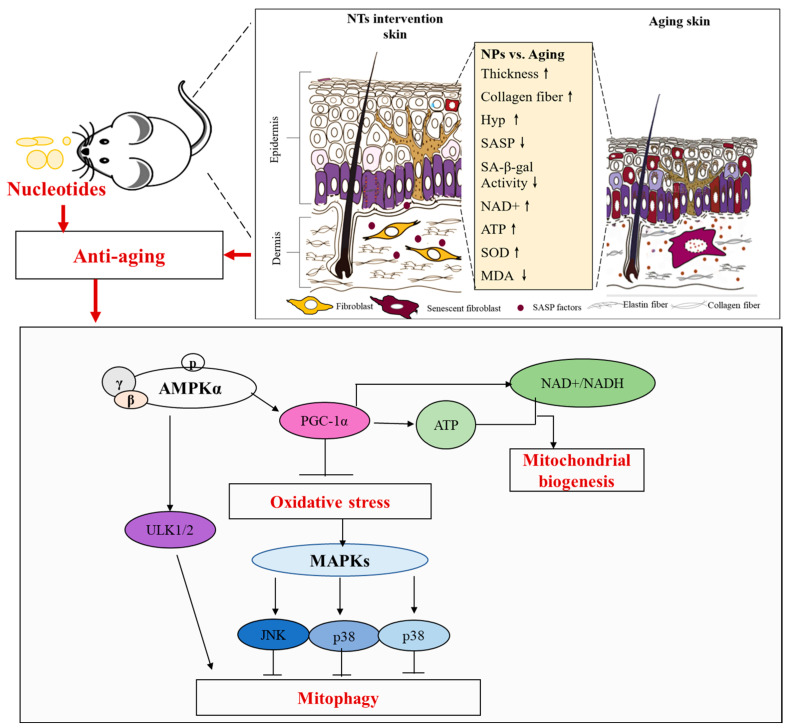
The anti-aging effects of NTs and their possible mechanism in skin tissue.

For autophagy, AMPK is a trigger in a double-pronged mechanism of directly activating ULK1/2 for mitophagy and inhibiting the suppressive effect of mTORC1 complex1 on ULK1/2 [72,73]. This study found NTs could significantly increase the expression of ULK1/2, which speculated that NTs promote autophagy as well as mitochondrial biogenesis. Interestingly, AMPK has the ability to induce the acute degradation of dysfunctional mitochondria via the ULK1/2-dependent activation of mitophagy, in addition to promoting the generation of new mitochondria through PGC-1α-mediated transcription [74]. Based on the dual mechanism of autophagy, our study identified that NTs may enhance autophagy via the AMPK pathway. However, the article did not specify whether the autophagy promoted by neurotrophins was mitochondrial autophagy or general autophagy. The potential mechanism by which neurotrophins exert their anti-aging effects is detailed in Figure 7. On the one hand, NTs could activate the AMPK pathway to a certain extent, thereby upregulating ULK2 expression leading to the promotion of autophagy; on the other hand, NTs regulate the generation of NAD^+^ through the AMPK-PCG-1a pathway, improve mitochondrial biogenesis, reduce oxidative stress, and further inhibit the activating of p38 pathway, and subsequently reduce the expression of *p*-JNK and *p*-ERK1/2, and thus activating autophagy from another pathway. Ultimately, NTs demonstrate anti-aging properties via the aforementioned pathways.

In summary, our results suggest that exogenous NTs improve both the morphology and the aging phenotype of ageing mice, due to decreased oxidative stress, improved mitochondrial function and the promotion of autophagy. The findings of this research suggest that the regulatory mechanisms involve the AMPK pathway and MAPK pathway. Further investigation into the specific molecular mechanisms, in conjunction with an exploration of the biological processes of NTs, is necessary to fully elucidate the NT-promoted autophagy mechanism. Additionally, we have confirmed the signaling pathway associated with prior research. Future research should encompass comprehensive multi-omics studies to explore a broader range of signaling pathways while taking into account the specificity of histiocytes. Additionally, this study has shown the anti-testicular aging impact of NTs in animal models, a finding that warrants further validation through population-based studies.

## 5. Conclusions

Our study demonstrates that supplementation with NTs significantly enhances the anti-aging properties. This improvement is likely attributable to increased autophagy levels and enhanced mitochondrial biogenetic function, achieved through the activation of the AMPK pathway and inhibition of the MAPK pathway.

## Data Availability

The data presented in this study are available on request from the corresponding author. The data are not publicly available due to privacy.
